# Assessment of Prickly Pear Fruit Peel Mucilage in Form of Gel as a Green Coagulant for the Tertiary Treatment of Domestic Wastewater

**DOI:** 10.3390/gels9090723

**Published:** 2023-09-06

**Authors:** María Carolina Otálora, Andrea Wilches-Torres, Carlos Rafael Lara, Jaime Díaz-Gómez, Jovanny A. Gómez Castaño, Gabriel Ricardo Cifuentes

**Affiliations:** 1 Grupo de Investigación en Ciencias Básicas (NÚCLEO), Facultad de Ciencias e Ingeniería, Universidad de Boyacá, Tunja 150003, Colombia; andreawilches@uniboyaca.edu.co; 2 Grupo Gestión de Recursos Hídricos, Facultad de Ciencias e Ingeniería, Universidad de Boyacá, Tunja 150003, Colombia; carlara@uniboyaca.edu.co (C.R.L.); jaimediaz@uniboyaca.edu.co (J.D.-G.); 3 Grupo Química-Física Molecular y Modelamiento Computacional (QUIMOL®), Escuela de Ciencias Químicas, Universidad Pedagógica y Tecnológica de Colombia, Avenida Central del Norte, Sede Tunja 150003, Colombia; jovanny.gomez@uptc.edu.co

**Keywords:** prickly pear (PP) fruit peel mucilage gel, novel coagulant, domestic wastewater treatment, turbidity and the color removal efficiency, flocs

## Abstract

The application of natural coagulants derived from food byproducts in domestic wastewater tertiary treatment, which contains a number of impurities as suspended colloidal particles, has a potential use as essential substitutes for traditional inorganic coagulants. These biomaterials are a sustainable and environmentally friendly alternative that can be used to improve water quality and human health. In this study, prickly pear (PP) fruit peel mucilage gel was evaluated as a novel coagulant for the tertiary stage of domestic wastewater treatment. Jar tests were performed on residual raw water at the inlet (influent) and outlet (effluent) of the tertiary wastewater treatment (constructed wetland) with a coagulant dose of 12 mg L^−1^ at a pH of 13. The efficiency of green (i.e., mucilage) and inorganic chemical (i.e., FeCl_3_) coagulants was compared on the basis of turbidity and color removal. The flocs produced by the coagulants were characterized structurally by FTIR spectroscopy and Zeta potential analysis and morphologically by scanning electron microscopy (SEM). The results showed that the turbidity and the color removal efficiency of the mucilage compared to the FeCl_3_ at the outlet of the treatment (effluent) were practically the same, reaching 94% turbidity and 85–87% color removal efficiency with both coagulants. The structure and morphology of the flocs generated by the coagulants showed a higher content of organic matter trapped in the flocs. The floc formation observed mechanisms were adsorption/bridging for mucilage and charge neutralization for FeCl_3_. The results of this study demonstrated that the PP mucilage green coagulant can be used to enhance the quality of treatment of domestic wastewater in an eco-friendly and biodegradable manner.

## 1. Introduction

Domestic wastewater is effluent contaminated by human activity in cities and must be reused and recycled safely in order to protect the environment [1]. The strategies for the reuse and recycling of such wastewater include three treatments, where secondary treatment is related to the transformation and degradation of organic matter, with the latter represented by the parameters of biochemical oxygen demand (BOD), chemical oxygen demand (COD), and total organic carbon (TOC). Because of this transformation, various forms of nutrients, especially phosphorus and nitrogen, are generally released into the effluent [2]. The reduction of these nutrients in the effluents is carried out by tertiary treatment [3]. There are numerous methods of tertiary treatment, and they include reverse incineration (a complete and direct process), adsorption (requiring fewer pretreatment processes), membrane separation (no sludge generation), and advanced oxidation processes (fast oxidation rate; no production of sludge) such as catalytic ozonation, Fenton and electrochemical oxidation, as well as coagulation [4], with the latter being widely used due to its high efficiency and its easy application [5].

In particular, the use of traditional inorganic salts based on metals, such as aluminum (e.g., Al_2_(SO_4_)_3_ and AlCl_3_), iron (e.g., Fe_2_(SO_4_)_3_ and FeCl_3_), and titanium (e.g., TiCl), has been implemented for the coagulation/flocculation process in contaminated wastewater effluents [3]. Despite the proven efficiency of inorganic coagulants, these coagulants have drawbacks associated with the environment and human health [3,6]. In contrast, natural or green coagulants represent renewable (i.e., feedstock can be obtained easily) and biodegradable options that are not only nontoxic to humans and the ecosystem but also relatively cost-effective (i.e., have a lower sludge volume and a lower treatment cost) [7].

Numerous studies agree that natural or green coagulants have technical and economic feasibility, which places them in favorable contrast to chemical coagulants. That is to say, the use of inorganic coagulants generates large amounts of sludge that require additional removal techniques before disposal, and it thereby increases the treatment cost compared to green coagulants. The latter, despite requiring compounds for pH conditioning, reduces the amount of sludge generated, and, once stabilized through anaerobic digestion, this sludge can also be used as biosolids for soil conditioning or fertilizer as well as valuable biogas for electricity generation. Moreover, biocoagulants have a very good ability to increase floc size, which promotes their efficacy in the treatment of wastewater, thereby reducing the cost of both production and the treatment of the wastewater [8,9,10,11,12].

In recent years, the valorization of Cactaceae family fruit processing byproducts (such as peels, which are generally disposed of as waste) has drawn the attention of researchers due to the fact that these byproducts could lead to the development of sustainable products, such as green coagulants for wastewater treatment, based on the concepts of a circular economy and a bioeconomy [13]. These fruit peels are characterized by their ability to exude a transparent gelatinous substance with exceptional water-holding and structuring (gel-forming) capacity at low concentrations, which is defined as mucilage [13]. This mucilage is a heteropolysaccharide of an anionic polyelectrolyte nature, with an average molecular weight of 0.44 KDa, and it primarily contains galacturonic acid, which is a molecule responsible for destabilizing colloidal suspended particles and forming flocs through a coagulation mechanism by adsorption and bridging. This is an interesting property for water treatment due to the high performance of these particles in the removal of turbidity and color [13,14].

Few green coagulants have been used in domestic wastewater treatment. However, Vega Andrade et al. [5] demonstrated that Moringa oleifera seed aqueous extract is a potential substitute for alum to be used as a coagulant for domestic wastewater tertiary treatment. Likewise, Barreto et al. [15] determined that coffee mucilages are better flocculants than avocado seeds and that they can be used to effectively remove turbidity and color in the primary treatment of domestic wastewater. Recently, we reported the optimal dosage of prickly pear fruit peel mucilage as a natural coagulant to be 12 mg L^−1^ at a pH of 13 in order to remove turbidity and remove color from synthetic turbid water, i.e., with realistic wastewater characteristics (a pH of 7.85 ± 0.09 and a conductivity of 147 ± 0.01 µS/cm), compared with ferric chloride (FeCl_3_) as a traditional inorganic coagulant [14]. However, the abovementioned biocoagulant can be used at a pH between 8 and 13 in order to avoid the addition of chemicals that allow the pH to be adjusted to neutral prior to discharge.

This study aimed to evaluate the efficacy of prickly pear (PP) fruit peel mucilage in the form of a gel as a green coagulant for the tertiary treatment of domestic wastewater as an alternative to conventional ferric chloride. The flocs obtained from the treatment of the water samples collected at the inlet (influent) and the outlet (effluent) of the constructed wetland were structurally analyzed using Fourier transform infrared spectroscopy (FTIR) and zeta potential analysis, and they were morphologically analyzed using scanning electron microscopy (SEM). The characterization of the flocs allowed the establishment of the predominant coagulation mechanism according to the type of coagulant used.

## 2. Results and Discussion

### 2.1. Study of the Efficiency of Mucilaginous PP Coagulant in Turbidity and Color Removal

The effect of inorganic (FeCl_3_) and green (PP fruit peel mucilage gel) coagulants on the removal of turbidity and color in domestic wastewater collected at the inlet (influent) and at the outlet (effluent) of the constructed wetland (tertiary treatment) is summarized in Table 1.

The domestic wastewater without coagulation treatment collected at the inlet of the tertiary treatment (influent) presented high values of turbidity (88.00 ± 0.50 NTU) and color (671 ± 2.54), which indicate organic micropollutants that persist even after the secondary treatment. Although the tertiary treatment favors the decrease nutrients, organics and metal concentrations. The turbidity (66.67 ± 0.27 NTU) parameter measured in the effluent (at the outlet) still presented high values in relation to maximum permissible values (2–5 NTU) [16]. In cases such as this, implementation of the coagulation/flocculation process (a chemical method used to remove impurities from water) is a highly recommended alternative for treating this type of effluent. For instance, Vega Andrade et al. [5] reported that the use of coagulants in the tertiary treatment of domestic wastewater allows the destabilization of the colloids present, providing an effluent that is less harmful to the environment.

As shown in Table 2, both coagulants (i.e., FeCl_3_ and PP fruit peel mucilage gel) showed high turbidity (>94%) and color (>85%) removal capacities in the wastewater sample collected after tertiary treatment (effluent). The high color and turbidity removal capacity in effluent is due to destabilizing of colloidal particles by charge neutralization (using FeCl_3_) or by adsorption-bridging particles (using PP fruit peel mucilage) [17]. Choy et al. [18] show a schematic diagram of these coagulation mechanisms. This result shows a contributory effect of the combined removal between the tertiary and the coagulation/flocculation treatments. Similar behavior was observed by Vega Andrade et al. [5] when using Moringa oleifera seed aqueous extract as a potential substitute for alum in domestic wastewater tertiary treatment. In contrast, the removal of turbidity (66% ≤ removal capacity ≤ 61%) and color (39% ≤ removal capacity ≤ 24%) in the wastewater samples collected at the inlet of the tertiary treatment (influent) was lower for both coagulants, which was attributed to the greater amount of colloidal particles that were not removed during the secondary treatment [5].

### 2.2. Structural Analysis of the Flocs

**Figure 1:**
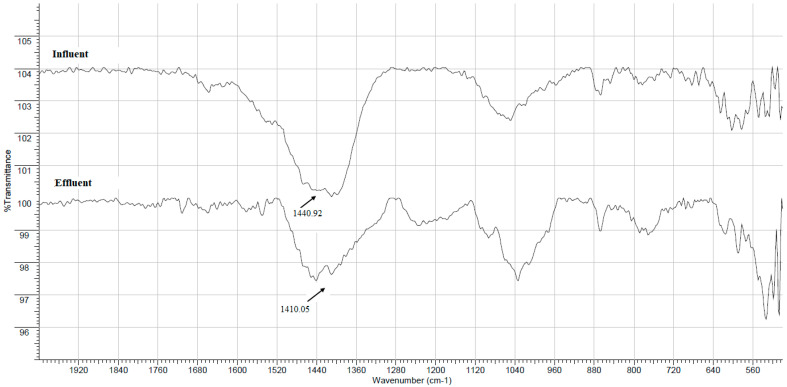
FTIR/ATR spectra in the range of 500 to 2000 cm^−1^ of the dried flocs formed using iron (III) chloride as a traditional chemical coagulant from the wastewater samples collected at the inlet (upper spectrum) and at the outlet (lower spectrum) of tertiary treatment.

**Figure 2:**
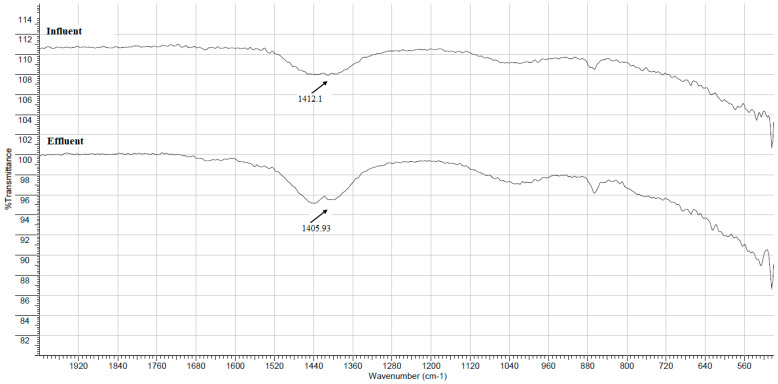
FTIR/ATR spectra in the range of 500 to 2000 cm^−1^ of the dried flocs formed using PP fruit peel mucilage as an alternative green coagulant from the wastewater samples collected at the inlet (upper spectrum) and the outlet (bottom spectrum) of the tertiary treatment.

TThe FTIR spectra of dried flocs formed using FeCl_3_ (chemical coagulant) and PP fruit peel mucilage (green coagulant) from the treatment of the influent and effluent are pre-sented in Figure 1 and Figure 2, respectively.

The infrared spectra of the flocs produced for the FeCl_3_ cationic coagulant (+9.55 ± 0.63 mV) in treatment of the influent and effluent present a broad band centered at 1440.92 cm^−1^ and 1410 cm^−1^, respectively, which can be attributed to the OCO bending vibration of the groups Fe-OCO-Fe formed from the deprotonation of the carboxylic groups (-COOH) of organic matter and the subsequent complexation of both oxygen atoms with Fe^3+^ ions [18]. This behavior is characteristic of a coagulation–flocculation process based on a charge neutralization mechanism, which involves the destabilization of the suspended particles using opposite-charged electric ions to attract them, and the particle charges then neutralize, which results in the electrostatic repulsion lessening or reaching a complete elimination [10]. Similar results were reported by Yue et al. [19], who used a macromolecular coagulant based on ferric salts and polyacrylic acid (Fe-PAA-1:1) for the removal of humic acids in wastewater treatment plants.

As shown in the FTIR spectra of Figure 2, the flocs formed from the treatment of the influent and effluent with PP fruit peel mucilage (anionic coagulant with −34.41 ± 1.66 mV) showed an absorption band within the range of 1600–1200 cm^−1^, which was attributed to the stretching vibration of the carboxyl functional groups of galacturonic acid present in the polysaccharide chains [13,20,21,22]. These functional groups possess H atoms that can make hydrogen bonds with other electronegative atoms bearing a lone pair of electrons in natural organic matter, and they can adsorb, in this way, the colloidal particles that are present in the wastewater [23]. This mechanism is consistent with the negative surface charge (i.e., zeta potential) measured in flocs obtained using natural mucilages [8]. For example, Wan et al. [24] reported a similar result in the flocs obtained by treating pond water from the oil–sand processing industry with *Opuntia ficus-indica* mucilage as the biocoagulant.

Additionally, a low-intensity absorption band with a wider contour (between 1657 and 1394 cm^−1^) is shown in the infrared spectra of the effluent compared to the spectra of the influent, which can be attributed to a higher number of solids adhering to both coagulants in the water collected at the outlet of the tertiary treatment during the coagulation–flocculation process.

**Figure 3:**
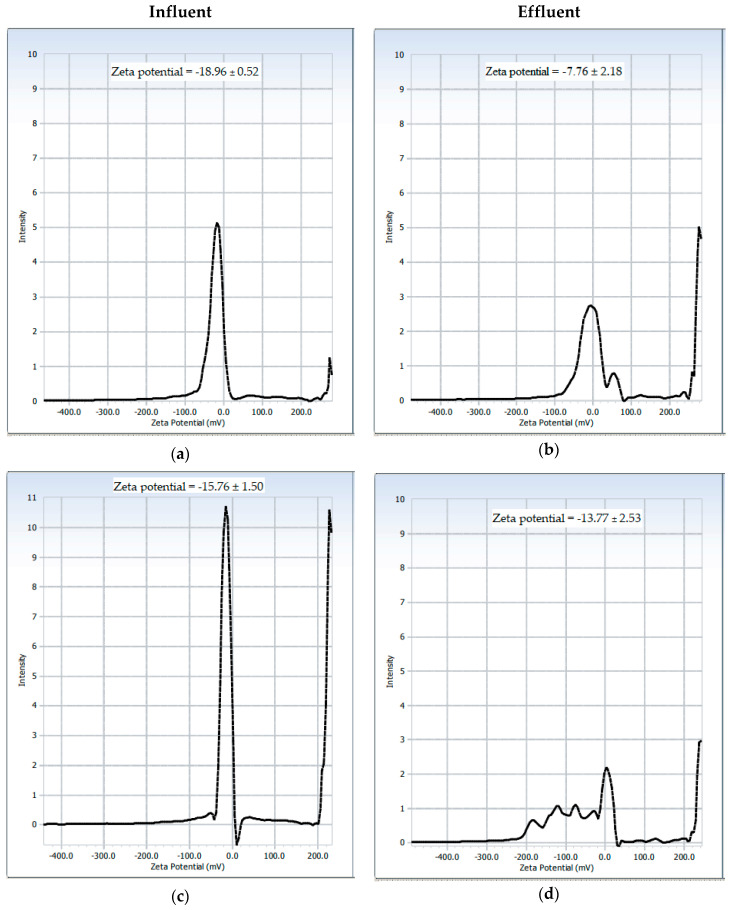
Zeta potential values for dry flocs formed from the coagulation/flocculation process of wastewater samples collected at the inlet (influent) and the outlet (effluent) of the tertiary treatment using FeCl_3_ (**a**,**b**) or PP fruit peel mucilage (**c**,**d**) as coagulating agents. Values were reported as the mean ± standard deviation (n = 3).

As shown in Figure 3, all of the zeta potential values measured in the flocs obtained with both the inorganic coagulant and the green coagulant are within the range of −20 mV to −5 mV, which is in agreement with the range established for the destabilization of colloidal particles in domestic wastewater [25].

The flocs obtained with both coagulants from the residual water sample collected at the outlet of the artificial wetland (effluent) presented zeta potential values closer to zero than those obtained from the samples collected at the inlet of the wetland (influent). This result is related to a higher rate of removal of contaminating macromolecules in the effluent both through coagulation mechanisms by adsorption and bridging between particles (coagulation with mucilage) and by charge neutralization mechanisms (coagulation with FeCl_3_) [8]. The lowest absolute zeta potential values obtained for the flocs of effluent indicated an efficient removal of organic matter with the established dose of coagulant (i.e., 12 mg L^−1^) and alkalinity (pH = 13). The greater removal of organic matter in the effluent also agreed with the greater removal capacity of turbidity (94.4%) and color (85.2–86.9%) that was obtained for this sample (Figure 3).

### 2.3. Morphological Analysis of Flocs

**Figure 4:**
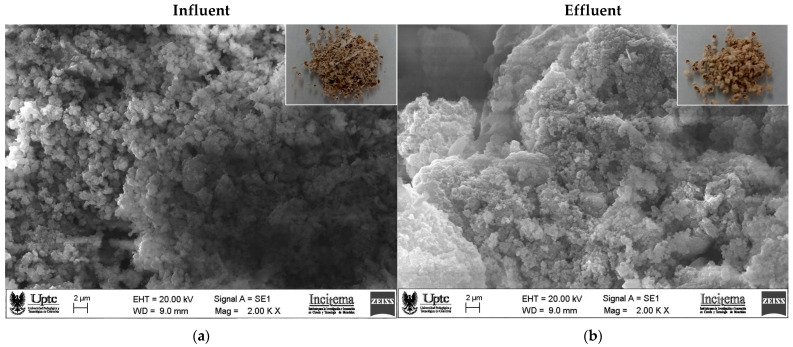

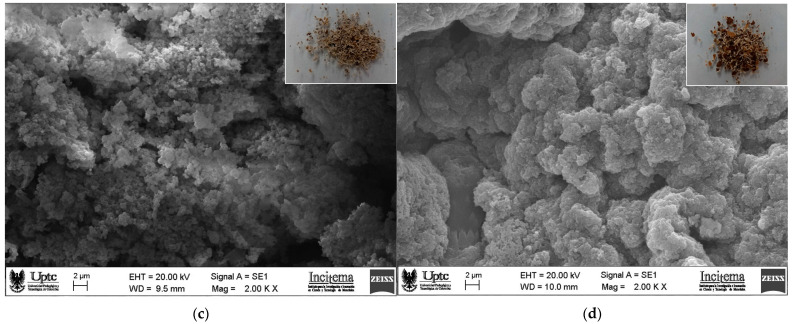
SEM micrograph images (with 2000× magnification) of the surface of dried flocs formed using iron (III) chloride (**a**,**b**) and PP fruit peel mucilage gel (**c**,**d**) as coagulants from wastewater influent (left column) and effluent (right column) samples. Photographs of the floccules are presented as insets at the top in each case.

The SEM micrographs of dried flocs formed after treatment of the influent and effluent with FeCl_3_ (chemical coagulant) and PP fruit peel mucilage gel (green coagulant) are presented in Figure 4.

The flocs generated after treating the influent with any of the two coagulants presented a cottony-looking morphology (Figure 4a,c). On the other hand, the morphology of the flocs formed from the effluent with both coagulants presented a coral reef-like appearance (Figure 4b,d) [26].

In particular, the inorganic coagulant applied to the influent (Figure 4a) generated light flocs with a regular shape (spheroid type) and a smooth surface, and this was in contrast to the effluent (Figure 4b), which generated thicker, amorphous flocs with cavities (i.e., scales) on the surface [27]. These morphologies could be attributed to the presence of aggregates formed through the interaction between the colloids that were suspended (with COO^−^ groups) and the Fe^3+^ ions. The ions neutralize the negative charges of the particles, thereby decreasing the electrostatic repulsion that favors coagulation and elimination of dissolved matter [10], and this is in agreement with the results obtained using FTIR and zeta potential analysis (Section 2.2 and Section 2.3). The presence of scales on the surface of the flocs obtained from the treatment of the effluent can be related to a greater number of colloidal particles captured compared to the number captured from the influent.

For its part, the green coagulant applied in the influent (Figure 4c) produced aggregated flocs that were irregular in shape (almost spherical) and stacked with deep empty spaces between the microflocs. In contrast, the effluent (Figure 4d) generated larger aggregated and dense flocs with irregular bulges. The morphology of both flocs obtained with the green coagulant can be attributed to the adsorption of colloidal particles with the reticular structure of the mucilage (a molecule with a porous and rough surface [10]) and the subsequent formation of hydrogen bonds, which leads to the formation of mucilage–colloid complexes [28], and this is in agreement with the results obtained using FTIR and zeta potential analysis (Section 2.2 and Section 2.3). The larger size of the floc and the presence of irregular bulges in the flocs of the effluent can be associated with a higher content of organic matter trapped in the flocs, a characteristic that directly measures the efficiency of the coagulation–flocculation process [29], and this is consistent with the results shown in Table 1. A similar morphology was observed for flocs formed with the leguminous plant Fenugreek for treating effluents from palm oil plants [30].

## 3. Conclusions

In this work, we have demonstrated the competitiveness of prickly pear peel mucilage as a primary coagulant for the removal of turbidity and color from real domestic wastewater before (influent) and after (effluent) tertiary treatment (i.e., wetlands) compared to a traditional inorganic coagulant such as FeCl_3_. The removal efficiency of turbidity (>94%) and color (>85%) in the effluent provided by PP fruit peel mucilage gel was almost the same as that of FeCl_3_. The structure and morphology of the flocs of the effluent generated with both coagulants revealed a higher content of organic matter trapped in the flocs both through adsorption/bridging coagulation mechanisms (using mucilage) and through charge neutralization (using FeCl_3_) compared to the content in the influent. This research has demonstrated that green coagulants can also be used to improve the quality of anaerobic effluents. However, the application of this biocoagulant in wastewater treatment has some limitations, such as financial (i.e., production, extraction, purification, and storage), market awareness, and regulatory approval constraints. Finally, in order to potentiate the use of this green coagulant, future research should focus on the effects of the mucilage on effluents with broad pH values > 10, such as effluents from the leather and textile industries.

## 4. Materials and Methods

### 4.1. Domestic Wastewater Tertiary Treatment Samples

**Figure 5:**
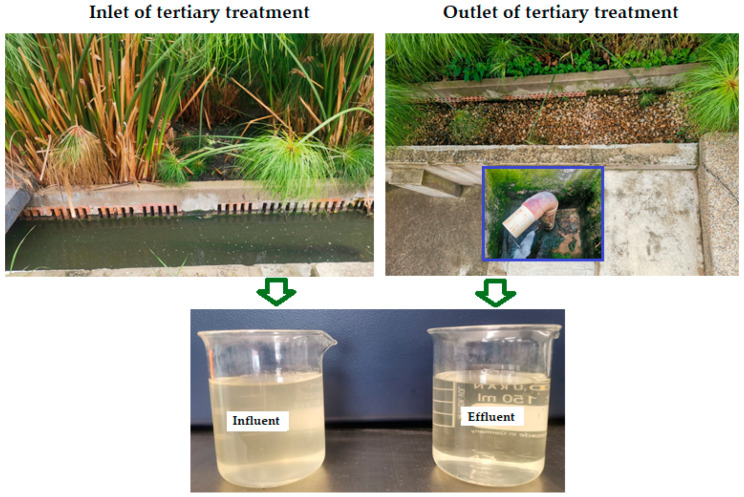
Selected photographs of raw wastewater collected at the inlet (**top-left**/influent) and outlet (**top-right**/effluent) of the tertiary treatment water level control structure.

Domestic wastewater samples were collected at the inlet and the outlet of the subsurface horizontal-flow artificial wetland (i.e., tertiary treatment) of the Universidad de Boyacá wastewater treatment plant located in the city of Tunja (5°32′7″ N and 73°22′04″ W), Colombia, at an altitude of 2750 AMSL (above mean sea level). The wastewater samples collected at the input (influent) and the output (effluent) of the constructed wetland (Figure 5) were physicochemically characterized according to the standardized methods prescribed by the APHA [31], as shown in Table 2. The turbidity and color parameters were determined using a turbidimeter (2100Q, HACH, Loveland, CO, USA) and a spectrophotometer (Hach DR 2800, USA), respectively. Turbidity and color are the most challenging factors in the effective treatment of wastewater with green coagulants [10].

The average temperature of the influent and the effluent was 19 ± 0.4 °C. The constructed wetland (length: 14 m; width: 4 m; slope: 0.5%) was implemented as a post-treatment unit for a 32 m^3^ UASB (upflow anaerobic sludge blanket) reactor for the treatment of domestic wastewater. This wetland was built in concrete, waterproofed with a geomembrane, and planted with *Typha domingensis*. The influence of plants (*T. domingensis*) on wastewater quality was demonstrated by an increase in the dissolved oxygen concentration from 0.0 to 1.0 mg L^−1^ in the wetland effluent. As a support medium in the wetland, gravel with a granulometry between 9.5 and 19 mm was used. The water level was 0.6 m, and the support media height was 0.7 m. The applied organic load was 8 g BOD/m^2^ d, and the hydraulic surface load was 0.03 m^3^/m^2^·d. The water level within the wetland was kept to 0.1 m below the surface using a water level control structure. The two samples were collected between September and October during peak hours, determined by high flow (8:00 am to 9:00 am), and were immediately transported to the environmental analysis laboratory.

### 4.2. Mucilaginous and Inorganic Coagulants

Mucilaginous coagulant (i.e., biocoagulant) was prepared as described by Otálora et al. [13]. In brief, hydrated prickly pear fruit peels were manually squeezed until a highly viscous gel was obtained. To the pressed gel, 95% ethanol was added in a ratio of 3:1 (ethanol:gel), and the mixture was kept until the formation of a milky white supernatant. This gelatinous supernatant, corresponding to the mucilage, was dried at 50 °C for 3 h. The dried mucilaginous material was reconstituted in distilled water, adapting a gel-like appearance, and it was used as a biocoagulant, as described below.

The biocoagulant stock solution was prepared following the procedure previously reported by us [14]. The stock solution of the inorganic coagulant was prepared by dissolving 12 g of ferric chloride (FeCl_3_·6H_2_O) in 1 L of distilled water at room temperature. Each coagulant was added separately to the wastewater samples in a dose of 12 mg L^−1^ at a pH of 13, and this was achieved with the addition of NaOH 1.0 M, according to the best conditions that we previously established for the promotion of an efficient coagulation/flocculation process [14].

### 4.3. Coagulation/Flocculation Process

**Figure 6:**
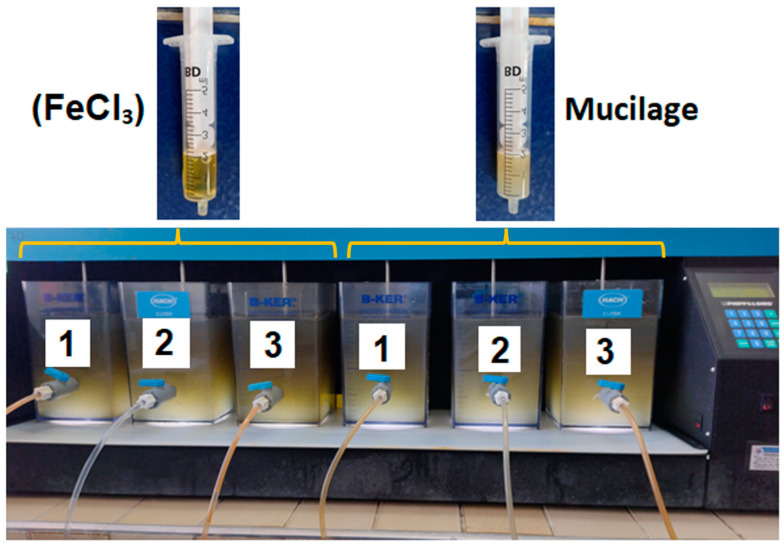
Photograph of the experimental setup used for the coagulation process of raw wastewater samples collected at the inlet and the outlet of tertiary treatment using FeCl_3_ and PP fruit peel mucilage gel as coagulants [13]. Labels 1 to 3 refer to the measurements performed in triplicate in each case.

To evaluate the turbidity and color removal capacities in wastewater influent (wetland inlet) and wastewater effluent (wetland outlet) exerted by the green coagulant (PP mucilage) and by the traditional inorganic coagulant (iron III chloride), a jar-test with six containers (2000 mL) (Phipps & Bird PB-900, Richmond, VA, USA) was used. This equipment (Figure 6) has an automatic controller that allows a fast mix at a speed of 100 rpm for 1 min to be programmed in order to reduce the natural repulsion between particles, and then a slow mix at 70 rpm for 20 min to be programmed in order to promote the grouping of particles in flocs. The mixtures were allowed to settle for 20 min before their quality analysis (i.e., turbidity and color), separation, and collection of the supernatant (i.e., flocs) (Figure 6). This flocculated material was dried in an oven at 50 °C for 24 h, and it was characterized structurally and morphologically.

### 4.4. Floc Characterization

#### 4.4.1. Flocs Structure

To identify chemical interactions between PP fruit peel mucilage, or FeCl_3,_ and colloidal particles during the coagulation process, Fourier transform infrared (FTIR) spectroscopy and zeta potential analysis were used. For this, a Bruker Alpha ECO-ATR FTIR spectrophotometer (Bruker, Germany) with a spectral range of 2000 to 500 cm^−1^ and a resolution of 4 cm^−1^ was used. The zeta potential was recorded using a NanoPlusTM 3 particle size zeta potential analyzer (Norcross, GA, USA) [13].

#### 4.4.2. Floc Morphology

To help elucidate the possible coagulation mechanism that occurred between the coagulants (mucilage or FeCl_3_) and the suspended solids, the surface morphology of the flocs was analyzed using scanning electron microscopy (SEM) using an EVO MA 10—Carl Zeiss (Oberkochen, Germany) operating at 20 kV. All samples were coated using gold–palladium sputtering before examination.

### 4.5. Statistical Analysis

The quality parameters (i.e., turbidity and color) of the tertiary treatment domestic wastewater samples and the zeta potential values of the flocs formed after the coagulation/flocculation process with each coagulant were processed using the analysis of variance (ANOVA) followed by Fisher’s test (*p* < 0.05). Values were reported as the mean ± standard deviation (*n* = 3).

## Figures and Tables

**Table 1:** Turbidity and color parameters and removal efficiency [%] in domestic wastewater collected at the inlet (influent) and the outlet (effluent) of the tertiary treatment stage (constructed wetland) before and after coagulation using FeCl_3_ and PP fruit peel mucilage gel as coagulants.

Parameter	Before Coagulation Process		After Coagulation Process			
	Influent	Effluent	Influent		Effluent	
			FeCl_3_	Mucilage	FeCl_3_	Mucilage
Turbidity (NTU)	88.00 ± 0.50	66.67 ± 0.27	29.50 ± 0.86 ^b^	34.26 ± 0.68 ^a^	3.76 ± 0.30 ^c^	3.76 ± 0.15 ^c^
Color (TCU)	671 ± 2.54	590 ± 3.12	412 ± 22.27 ^b^	509 ± 23.11 ^a^	77.33 ± 3.51 ^d^	87.00 ± 8.18 ^c^
Turbidity removal [%]	-	-	66.47 ± 0.98 ^b^	61.06 ± 0.77 ^b^	94.35 ± 0.45 ^a^	94.35 ± 0.22 ^a^
Color removal [%]	-	-	38.59 ± 3.31 ^b^	24.09 ± 3.44 ^c^	86.89 ± 0.59 ^a^	85.25 ± 1.38 ^a^

The means of three replicates ± standard deviation with different letters in the same row for each parameter indicate significant differences (*p* < 0.05) between samples, and the letter “^a^” corresponds to the higher value.

**Table 2:** Physicochemical parameters of the wastewater samples collected at the inlet and outlet of the constructed wetland.

Wastewater Sample	pH	Conductivity [in µS/cm]	COD [in mg L^−1^]	COD Filtrated * [in mg L^−1^]	Turbidity (NTU)	Color (TCU)	BOD (mg/L)
Inlet (influent)	7.32 ± 0.1 ^a^	1397 ± 32.52 ^b^	221 ± 34 ^a^	182 ± 11 ^a^	88.00 ± 0.50 ^a^	671 ± 2.54 ^a^	171 ± 10 ^a^
Outlet (effluent)	7.35 ± 0.1 ^a^	1558 ± 36.06 ^a^	35 ± 3 ^b^	13 ± 2 ^b^	66.67 ± 0.27 ^b^	590 ± 3.12 ^b^	15 ± 3 ^b^

* Filtered through a 0.45 µm Millipore filter. The means of three replicates ± standard deviation with a different letter in the same column indicate a significant difference (*p* < 0.05) between the samples, and the letter “^a^” corresponds to the higher value.

## Data Availability

The data presented in this study are available on request from the corresponding authors.
